# Supplementary material for: Sporothrix spp. Biofilms Impact in the Zoonotic Transmission Route: Feline Claws Associated Biofilms, Itraconazole Tolerance, and Potential Repurposing for Miltefosine
Source: Pathogens. 2022 Feb 3;11(2):206. doi: 10.3390/pathogens11020206 (PMC8880041; doi:10.3390/pathogens11020206)
Supplement: Supplementary file 1 [file pathogens-11-00206-s001.zip › pathogens-1553524-SI.pdf]

Supplementary Table S1. Susceptibility of planktonic yeast cells of *S. brasiliensis* and *S. schenckii.* Concentrations in µg/ml.

	MIC_{50} LR	MIC_{50}	MIC_{90}	MFC
***S. brasiliensis* ATCC 4823**				
Itraconazole	0.025	< 0.03 - 0.03	0.125 - 0.25	> 16
Amphotericin B	0.069	0.06 - 0.125	0.125	< 0.5
Miltefosine	0.313	0.5	2 - 4	2 - 1
***S. brasiliensis* ATCC 4824**				
Itraconazole	0.098	0.03 - 0.125	0.125 - 0.25	> 16
Amphotericin B	0.394	0.06	0.06 - 0.125	< 0.5
Miltefosine	0.959	0.25 - 0.5	0.5 - 1	1
***S. brasiliensis* ss245**				
Itraconazole	0.546	0.125 - 0.25	1 - 2	> 16
Amphotericin B	2.891	0.125	0.25	< 0.5
Miltefosine	3.051	0.25 - 1	0.5 - 2	1
***S. schenckii* ATCC 32286**				
Itraconazole	0.079	0.06 - 0.25	0.25 - 2	> 16
Amphotericin B	0.125	0.125	0.25	1 - < 0.5
Miltefosine	0.667	1	2	2

LR: MIC_{50} calculated by Linear Regression.

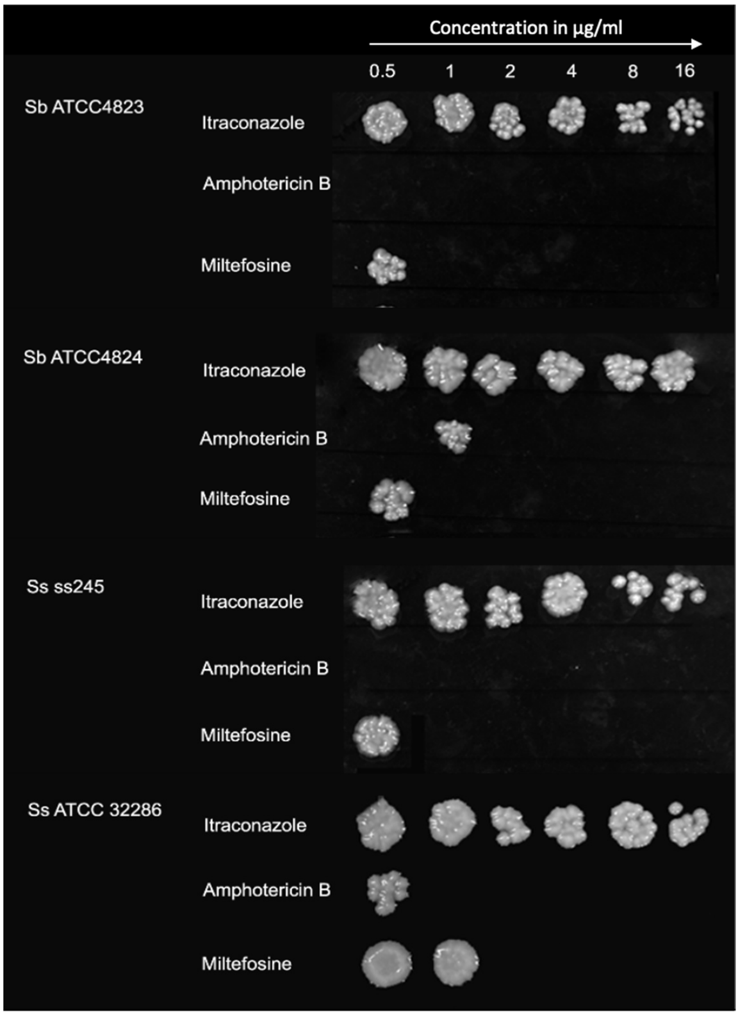

Supplementary Figure S1. Spotting inhibitory concentrations from the MIC assay shows a potential fungicidal profile for miltefosine.
